# Three-Phase Motor Inverter and Current Sensing GaN Power IC

**DOI:** 10.3390/s23146512

**Published:** 2023-07-19

**Authors:** Stefan Mönch, Richard Reiner, Michael Basler, Daniel Grieshaber, Fouad Benkhelifa, Patrick Waltereit, Rüdiger Quay

**Affiliations:** 1 Fraunhofer Institute for Applied Solid State Physics IAF, Tullastr. 72, 79108 Freiburg, Germany; 2 Department of Sustainable Systems Engineering (INATECH), University of Freiburg, 79110 Freiburg, Germany

**Keywords:** current measurement, power integrated circuits, gallium nitride, HEMTs, AC motor drives, three-phase DC–AC inverters, wide-bandgap semiconductors, current mirrors, brushless motors, equivalent circuits, substrates

## Abstract

A three-phase GaN-based motor inverter IC with three integrated phase current mirror sensors (sense-FETs or sense-HEMTs, 1200:1 ratio), a temperature sensor, and an amplifier is presented and experimentally operated. The three low-side currents are read out by virtual grounding transimpedance amplifiers. A modified summed DC current readout circuit using only one amplifier is also discussed. During continuous 24 V motor operation with space-vector pulse width modulation (SVPWM), the sensor signal is measured and a bidirectional measurement capability is verified. The measured risetime of the sensor signal is 51 ns, indicating around 7 MHz bandwidth (without intentional optimization for high bandwidth). The IC is operated up to 32 V on DC-biased semi-floating substrate to limit negative static back-gating of the high-side transistors to around −7% of the DC-link voltage. Analysis of the capacitive coupling from the three switch-nodes to the substrate is calculated for SVPWM based on capacitance measurement, resulting in four discrete semi-floating substrate voltage levels, which is experimentally verified. Integrated advanced power converter topologies with sensors improve the power density of power electronics applications, such as for low-voltage motor drive.

## 1. Introduction

### 1.1. GaN Power Converter ICs and Current Sensing

Gallium nitride (GaN) transistors are ideal for low-voltage (12 V to 48 V) motor drives, since they increase the efficiency and compactness of the system [1]. A three-phase inverter consists of six transistors to generate three AC output voltages from one DC-link input. The lateral GaN-on-Si technology allows monolithic integration of power and logic devices on one chip. However, commercially there are only low-voltage (<100 V) half-bridge GaN ICs available [2], which still requires three ICs to realize a three-phase topology [3]. GaN-based monolithic high-voltage converter topologies that include high-side devices are not yet commercialized as single-chip solutions because of substrate biasing issues [4]. These substrate-biasing issues include (as listed in [5]) capacitive coupling [6], cross-talk [7] through the conductive Si substrate, static (back-gating related) and dynamic (trap-related) on-resistance increase [7,8,9,10,11,12,13,14,15,16], threshold-voltage shift [17], buffer leakage [18], and changed gate charge [6]. GaN-on-SOI [19] or GaN-on-Si-based pn-junction isolation [20] might be a solution to avoid substrate effects at high voltages, but have increased device capacitances [21] from the buried oxide [19] or pn-junction depletion region compared to discrete circuits. However, with a simpler and lower-cost fabrication process, GaN-on-Si circuits are still a desired solution. Several half-bridge GaN ICs have been reported [22,23,24], but only few advanced converter topologies have been demonstrated as ICs so far: a three-phase inverter bridge was operated in 2009 [25], an AC–AC matrix inverter in 2014 [26], a multilevel converter in 2017 [27], and a half-bridge with secondary side switches in 2022 [28]. Further advanced monolithic integrated GaN power converters, devices, and building blocks in the GaN-on-Si power integration technology by Fraunhofer IAF, which is used in this work, are reviewed in [5,23,29], respectively. The control of motor drives typically is based on measurement of the three phase currents. To simplify the current sensors, it is also possible to just measure the three phase leg currents in the low-side transistors in each of the three phases, referenced to a common negative (ground) terminal. Furthermore, advanced motor control methods exist that only require measurement of two phase leg currents, or even just the sum of all three phase leg currents. The current measurement is typically realized by shunt resistors and operational amplifiers. However, the insertion of a shunt resistor adds additional conduction loss, reducing the efficiency of the inverter, and also is an intrusive method. This means that, to measure the phase leg currents, the electrical connection (trace) between the phase legs (source terminals of the low side transistors) and the DC-link decoupling capacitor has to be cut in order to insert a shunt. The remaining circuit layout (e.g., on a printed circuit board) has increased parasitic inductance in the critical (switched) current loops, possibly deteriorating the switching behavior and loss. Furthermore, for monolithic three-phase ICs, the insertion of three external shunts might not be possible at all, for example, if the common negative DC-link terminal is electrically connected already in the IC. To avoid the need for series-insertion of sensor in the critical loop (series connection of the IC, shunt and DC-link capacitor), a parallel-insertion of a current mirror transistor also enables the measurement of the main transistor current. The parallel connection of a sense transistor (sense-FET, also called sense-HEMT or sense-GaN for GaN HEMTs [30]) does not require the critical loop to be cut, and can be also monolithically integrated with the GaN IC. GaN-based sense-FET ICs have been already demonstrated monolithically [31,32,33,34,35,36,37] and also by external wiring of several GaN devices [30,38] for single discrete transistors and used in half-bridges. This work focuses only on current-mirror sensing; other current sensing methods such as shunt-sensor [39,40,41,42,43] or hall-sensor [44] integration, as well as related magnetic flux concentrators [45] or magnetic sensors [46], were also demonstrated in GaN technologies. External current sensors or two-chip solutions (GaN and Si) are also often used and investigated for GaN power electronics [47,48,49,50,51,52,53,54,55,56,57]. State-of-the-art low-voltage GaN motor inverters today still use at least three half-bridge ICs and three sense resistors [3,58,59]. In silicon (Si), monolithic three-phase motor drive ICs with integrated current sensing and other functionality have been available for decades, for example, using a lateral insulated gate bipolar transistor (LIGBT) power IC technology [60]. Compared to discrete multi-chip semiconductor approaches, such highly integrated single-chip solutions can be packaged in one small package, and thus improve the power density for low-voltage motor drive applications. Such highly integrated functional (current sensing) and power (three-phase bridge) ICs have, however, not yet been demonstrated in a monolithic GaN power technology. This work now presents such a monolithic three-phase converter GaN IC with integrated sense-FET-based current sensing.

### 1.2. Structure of the Work

Section 2 (Methods) discusses the three-phase GaN ICs used in this work (Section 2.1), and the current sensor readout circuit, as well as the substrate biasing network (Section 2.2).

Section 3 (Results) presents the experimental setup (Section 3.1) and measurement results of the current sensor (Section 3.2) and substrate biasing (Section 3.3).

## 2. Materials and Methods

### 2.1. GaN IC Power Design and Characterization

**Figure 1:**
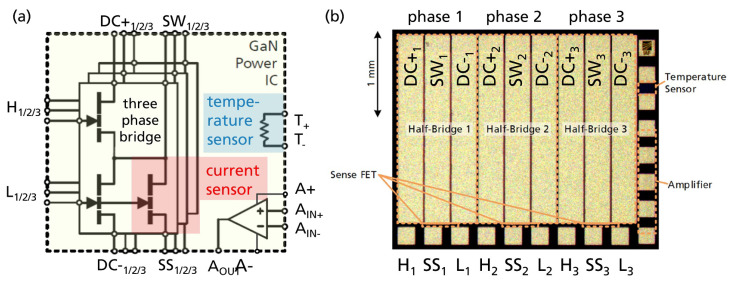
Three-phase GaN power IC with three integrated current mirror sensors, a temperature sensor, and amplifier: (**a**) Schematic. (**b**) Chip photo.

Figure 1 shows the schematic and layout of the GaN power IC. This work presents and uses a GaN-on-Si power IC which monolithically integrates a three-phase inverter topology, three current sensors, one temperature sensor, and an amplifier. An area-efficient matrix layout with interleaved low-/high-side half-bridge fingers (instead of side-by-side integration) is used, and the optimal design method is presented in [61]. Each power transistor has a gate width of 120 mm, and gate-to-drain distance of 1.5 µm, dimensioned for up to 48 V. In addition to an already-published three-phase GaN motor inverter IC [62], this work now also integrates three sense-HEMTs for current measurement, parallel to the low-side power transistors.

The drain (switch-node of the half-bridge) and gate are already connected on-chip with the main power transistors, such that only one additional bond pad per phase (the source of the sense-HEMTs) is required for external connections to readout circuits. The sense-HEMTs have a gate width of 100 µm, resulting in a gate width ratio and theoretical sense current ratio of 1200:1. One bond pad for each source of the sense-HEMTs forms the electrical interface to external readout circuits. The IC also integrates an isolated temperature sensor (resistive gold–metal meander trace [63], two bond pads), and a monolithic differential amplifier (five bond pads) as in [64]. The integrated one-stage differential amplifier is not used in this work, but can be extended to a multi-stage and high-gain integrated amplifier circuit as was experimentally demonstrated in [41] for current-sensing purposes.

**Figure 2:**
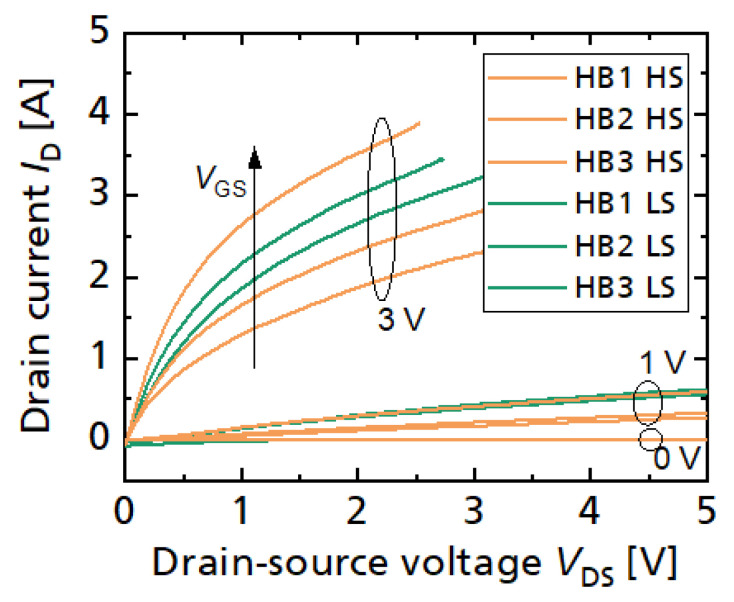
Measured output characteristic of the six integrated power transistors (250 mΩ each).

Figure 2 shows measured on-state characteristics, where each power transistor has around 250 mΩ on-state resistance. The GaN HEMTs are normally-off using a pGaN gate module [65]. The particular wafer run has an increased gate forward leakage currents, limiting the applicable gate-to-source voltage (for below 100 mA gate current) to around 3 V, but not further hindering device operation.

Using the same three-phase design and same chip size, lower on-resistances have also been achieved, e.g., 110 mΩ per transistor [61] (normally-off) or 60 mΩ in a normally-on wafer run with lower sheet resistance [62], which is a low area-specific on-resistance of 0.25 mΩ cm^2^ (in the active area).

### 2.2. Current Sensor Readout Circuits and Substrate Biasing Network

**Figure 3:**
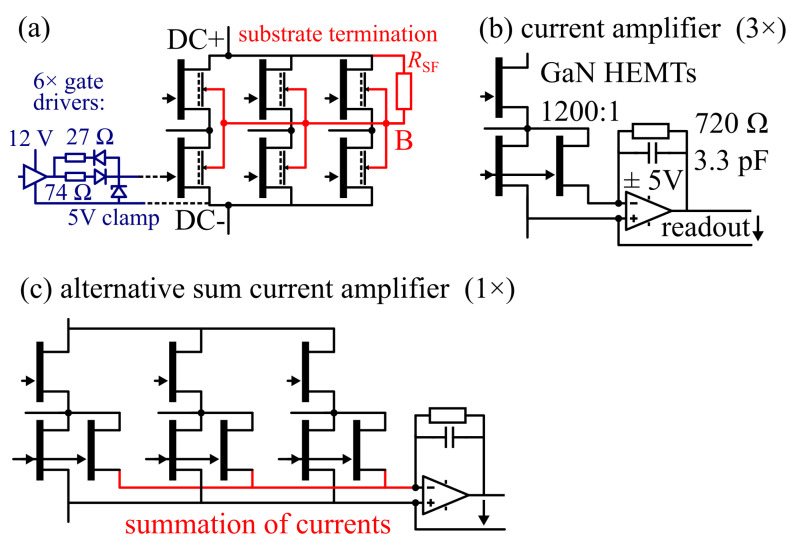
External circuitry: (**a**) Gate driver circuit adapted to motor evaluation board and substrate biasing network for positive dc+ biased semi-floating substrate termination. (**b**) Three transimpedance amplifiers in virtual grounding configuration. (**c**) Alternative one-amplifier summed current readout circuit.

The GaN IC is later operated in a motor drive evaluation board, where the one GaN IC replaces the six Si MOSFETs of the evaluation board. The 12 V gate driver of the demo board is reused and adapted to the 5 V gate voltage limit by the gate drive circuit shown in Figure 3a. As previously stated, the particular wafer run had excessive gate leakage. To limit the gate forward current during the on-state, a high 74 Ω gate resistor was used, which limits the gate forward current to around 100 mA. The transistor is thus controlled with an on-state gate current (similar to commercially available gate injection transistors) instead of a constant gate voltage. Since the three-phase circuit in the GaN-on-Si IC is operated on a common conductive Si substrate, the substrate-to-source voltage of each power transistor cannot be set to zero volt, as in discrete GaN power HEMTs, individually. This work uses a common positive (DC+) biased substrate *B* termination, which shifts the average substrate potential towards positive values, and avoids negative back-gating-related on-state resistance increase in high-side transistors during high-side on-state conditions. This semi-floating substrate termination for three-phase bridges was proposed and analyzed in [62], and was derived from semi-floating substrate termination of monolithic GaN-on-Si half-bridges [4,18,66,67,68]. Capacitive substrate coupling and the effect of substrate-biasing on back-gated on-resistance increase is further discussed later in Section 3.3.

**Figure 4:**
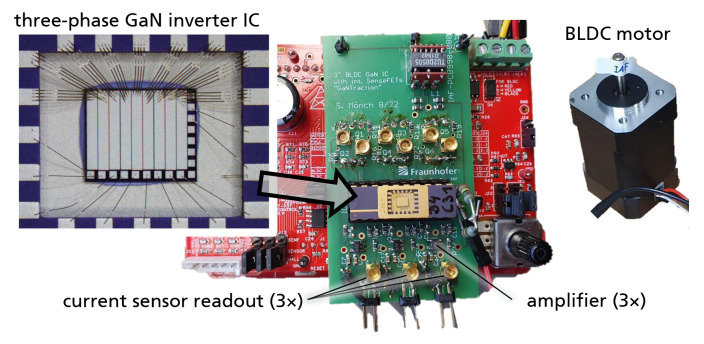
Experimental setup of three-phase GaN power IC (2.5×3 mm^2^) with three integrated current mirror sensors, bonded in package, and operated in motor drive evaluation board. The connectors allow reading out of the three amplified current signals.

Figure 3b shows the current sensor readout circuit used in this work, which consists of an external Si-based operational amplifier (TI OPA653) in transimpedance configuration (virtual grounding). The drain current of the main power transistor is mirrored (due to the same gate-to-drain geometry and intrinsic device structure, and same drain–source and gate–source voltages) and scaled down (factor 1200:1) in the sense transistor. The sense current causes a voltage drop on the feedback resistor (R=720Ω) which is also the sensor readout voltage. The theoretical current-to-voltage gain is 720/1200 V/A = 0.6 V/A, and the bipolar 5 V amplifier supply covers a current measurement range of −8…+8 A. A low-pass filter (C=3.3pF) is also used in the feedback. It should be mentioned that the sense-HEMT can reach the same switching speed as the main power transistors, and if the amplifier circuit is well optimized, theoretically, a very high bandwidth at >10 MHz with fast settling time could be reached. In the literature, GaN-based senseFETs with higher bandwidths (43 MHz [32] and 9.2 MHz (reported risetime 38 ns) [35]) have been published. Those published senseFET and readout circuit approaches (for example, integrating a burden resistor instead of, or in addition to, the external amplifier to reduce the parasitic loops between the sensor and amplifier) could be adapted to this work’s IC and then a higher bandwidth is expected. Future works can investigate a high-frequency design optimization of the critical loops between the power IC and the amplifier, in order to further improve the sensed signal quality (e.g., bandwidth, response time, overshoot) compared to this work. This readout circuit is realized three times, and the output voltage is measured in the later experiments through electrical connectors on the board (later marked in Figure 4).

The approach with three readout circuits of the three low-side phase currents enables current-control of three-phase motors, for example, as in the well-known three-shunt method. An alternative sensor readout configuration is shown in Figure 3c. Here, the three source terminals of the three sense HEMTs are connected and the summed sense current is input to only one transimpedance amplifier. This configuration simplifies the amplifier circuit (requiring only one amplifier, since the sense currents are summed up by the connection of the sense terminals). Such simplified readout circuits still allow control of three-phase motors, for example using a one-shunt control method, but would require only one additional current sense terminal for the complete power IC.

## 3. Results

### 3.1. Experimental Setups

Figure 4 shows the experimental setup for motor operation and current measurement. A motor drive evaluation board (CY8CKIT-037) is used, and the six Si MOSFETs are replaced through an adapter with the one GaN IC. The IC is soldered into a DIP20 package with conductive micro-silver sinter paste. A 24 V BLDC motor is connected to the board without additional mechanical load. The substrate biasing-resistor is 2 MΩ. The motor phase current, the three switch-node voltage, the semi-floating substrate voltage, and the sensor output signals are measured with an oscilloscope.

**Figure 5:**
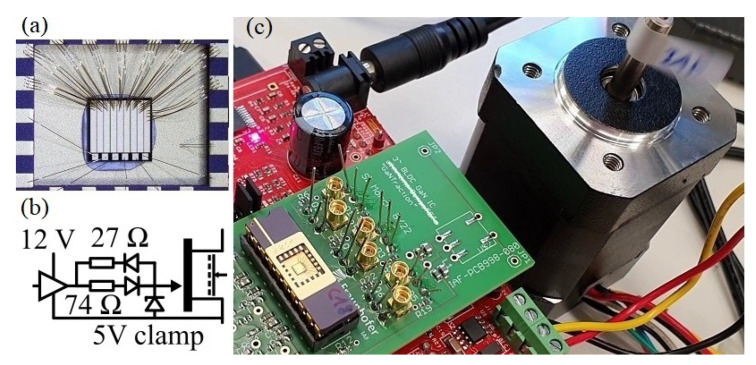
Simplified setup for substrate coupling analysis. (**a**) 2×2 mm^2^ monolithic three-phase motor inverter GaN IC (without current sensors) in DIP20 package. (**b**) Gate driver circuit. (**c**) Controlled by a motor evaluation board.

Figure 5 shows a similar second setup, which is later used for analyzing the substrate coupling effects. This second setup uses a simplified and more area-efficient IC (2×2 mm^2^) without current sensors, but the same substrate biasing network, such that almost the same substrate biasing effects occur as in the IC with current sensors.

The efficiency of the motor inverter was not measured. It should be mentioned that due to the limited available chip area on the multi-project-wafer run, only a small (2.5×3 mm^2^) chip was designed. The on-resistance of the six GaN transistors is, thus, significantly higher than the on-resistance of the Si-MOSFETs, which were initially on the motor evaluation board. A high efficiency can only be expected if the motor power-rating is matched to the inverter power-rating, which is not the case in this work. Since the motor is only operated with no additional mechanical load, the GaN IC still can provide the required driving power for the free-running motor operation. The complete IC could be scaled to higher gate widths in future in a production run, or a smaller motor could be driven in future to quantify the power conversion efficiency. In this work, however, the focus is on the three-phase operation and integrated current sensing.

### 3.2. Current Sensor Measurements

**Figure 6:**
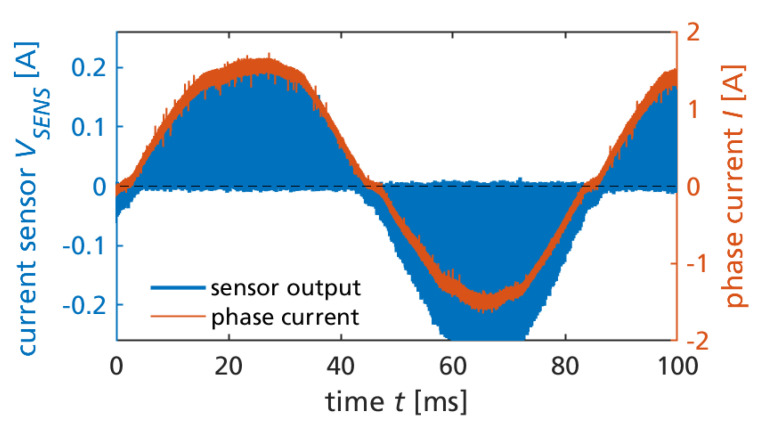
Measured current sensor output (after the transimpedance amplifier) and phase current.

The motor was operated at 24 V continuous constant-speed operation (around 12 Hz of the sinusoidal phase currents) with no mechanical load, resulting in peak currents of around 1.6 A. No additional heatsink was attached to the DIP package for the GaN ICs. Figure 6 shows one measured phase current and the respective current sensor’s output voltage $V_{\mathrm{SENS}}$. From the gate width ratio and transimpedance configuration, a theoretical sensitivity of 0.6 V/A was expected (see Section 2). The measured sensitivity, however, is systematically and significantly lower, between 0.13 V/A at positive phase currents, and 0.17 V/A at negative phase currents. As was previously mentioned, the particular wafer run had unstable gate characteristics, with increased gate forward leakage current, such that the gate–source voltages during on-state were lower than the gate driver supply voltages. One possible explanation is, that because the gate of the main transistor and the respective sense-HEMT was already connected on-chip (not allowing separate gate drive circuits), either of the transistors was not in full on-state, such that the assumed ideal current mirroring was not actually achieved. Furthermore, in forward and reverse conduction, the voltage drop across the gate–source distance (part of the total gate–drain channel length) has different signs. Therefore, the gate current-limited operation can cause a significant variation of the resulting gate–source voltage and, thus, on-state if the undesired gate-leakage does not equally scale between the senseFET and the main power transistor. This is a possible explanation for why the sensor gain differs by around 30% between the positive and negative current phases. In a previous work, a GaN half-bridge with integrated current-mirror sensors (similar to this work, but not in a three-phase configuration) from a more stable normally-off wafer run was experimentally characterized [64], and showed a good match of theoretical and measured current ratio. Thus, a higher sensor sensitivity, more close to the theoretical values, can be expected when fabricating future ICs with a more stable technology.

In Figure 6, the high switching frequency is not visible, but it is clearly visible that the sensor signal returns to zero within each switching cycle. This behavior is expected for the low-side current sensing, in contrast to a continuous phase-current sensing.

**Figure 7:**
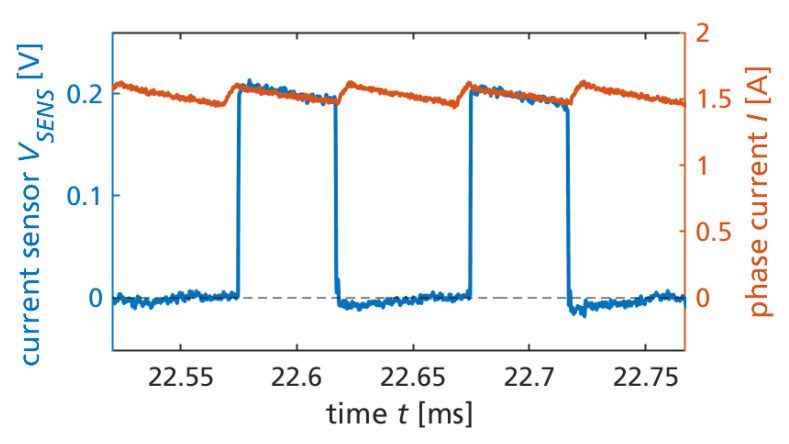
Measured current sensor signal and phase current during two switching cycles and continuous SVPWM motor control and operation.

Figure 7 shows a zoom into Figure 6 during two switching cycle times. The piecewise linear inductor current is approximately triangular in the particular operation point with no mechanical load. More precisely, the space vector pulse width modulation (SVPWM) switches all three phases, resulting in six states within a switching cycle. Furthermore, the particular implementation of the (conventional) SVPWM uses both available zero voltage states (all switches low or all switches high) alternately. Therefore, the sensor signal is available only with half the frequency compared to the phase current, which is clearly visible in the figure.

**Figure 8:**
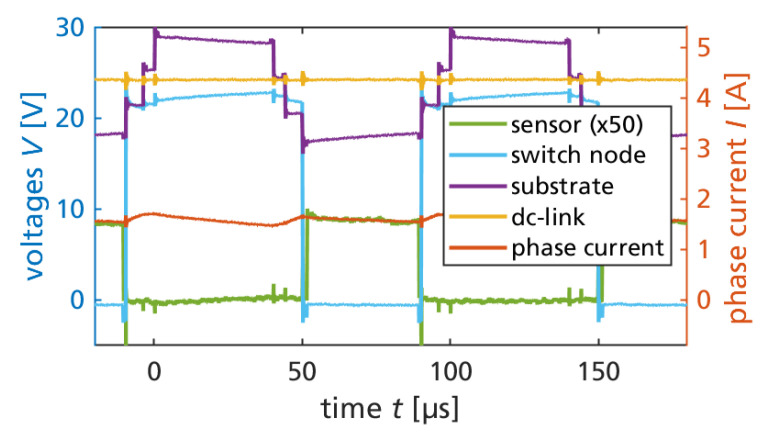
Measured voltages (sensor output, switch node, substrate, DC-link) and phase current during two switching cycles.

The operation of the monolithic GaN converter IC is also subject to substrate biasing. Figure 8 shows the (semi-floating, DC-biased) substrate voltage, switch node voltage, and 24 V DC-link voltage, simultaneously measured with the phase current and sensor signal during two switching cycles. The capacitive substrate biasing is later discussed in Section 3.3.

**Figure 9:**
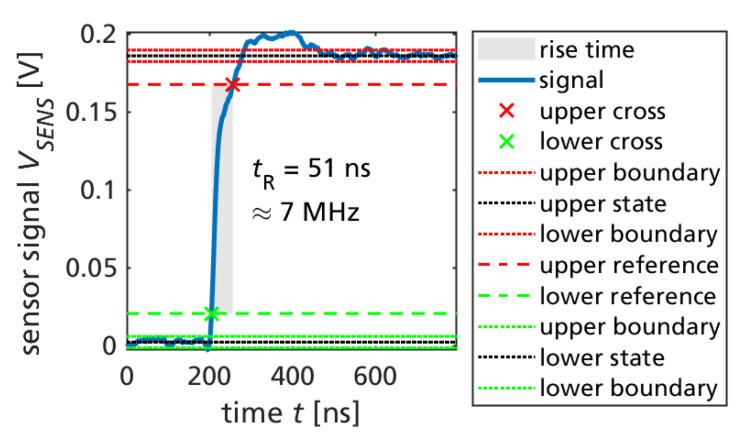
Measured risetime (10–90%) of 51 ns, indicating around 7 MHz bandwidth.

For one typical switching transition, the risetime of the sensor signal is extracted (between the 10% and 99% state levels of the sensor signal). Figure 9 shows the measured risetime of 51 ns. From this very fast risetime, around 7 MHz sensor bandwidth is estimated (from the approximation 0.35/51 ns). This makes the integrated current mirror sensing particularly useful for control of fast-switching GaN power converters. It should be mentioned that the readout circuit in this work was not yet optimized for a high bandwidth, and even faster risetime (close to the below 10 ns switching time of the power transistor) could be reached by future works.

### 3.3. Substrate Biasing Analysis during Motor Operation

Capacitive substrate biasing effects during operation of previously published three-phase motor inverter GaN ICs [25,26] were not yet experimentally analyzed. Theoretically, the effect of the three switch node voltages on the transient substrate voltage, resulting in four discrete substrate voltage levels (compared to two in half-bridges) was hypothesized in [62] but not yet verified. Both are now covered in this paper.

To focus on the capacitive coupling from the three-phase bridge, in this section, a simplified three-phase IC without sensors is used (see Figure 5), which is the exact same device as presented and characterized in [61] The analysis and measurements are carried out at 32 V and 24 V and are valid for both voltages (the substrate capacitances form capacitive voltage dividers coupled to the DC-link voltages), and the results are presented partly in voltage ratios (scaled to the DC-link voltage), which allows us to scale this work’s results to the desired voltage class (e.g., 24 V, 32 V, and 48 V).

**Figure 10:**
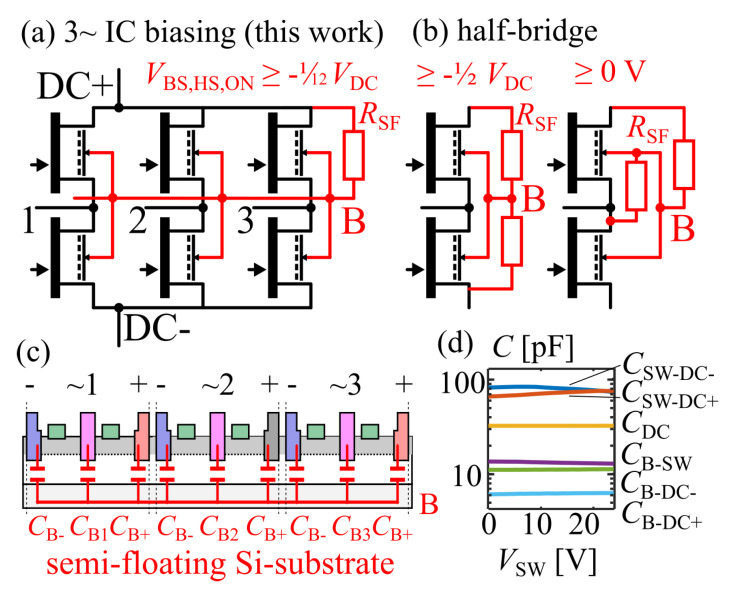
(**a**) Highly resistive substrate biasing to DC+ for limited negative back-gating (three-phase IC). (**b**) Half-bridge biasing, figures adapted from [66,69]. (**c**) Substrate capacitance equivalent circuit with three switched phases. (**d**) Measured capacitances (one half-bridge); the phase (switch-node) voltage is swept, keeping the DC-link at 24 V.

Capacitive substrate coupling is analyzed for the three-phase GaN IC with a positive DC-biased floating substrate (Figure 10a) with the equivalent substrate capacitances as shown in Figure 10c. This allows us to analyze the transient substrate voltage during three-phase space vector pulse width modulation (SVPWM) (details in [62]).

A semi-floating substrate termination is used in this work because it maintains an almost symmetrical switching behavior between the high-side and low-side devices, in contrast to a fixed substrate termination such as connection to one of the DC-link terminals or the switch node (analysis in [21]). Furthermore, the floating substrate slightly reduces the effective switch node capacitance compared to discrete half-bridge transistors with separate substrate-to-source terminations [21]. The positive biasing is used because it avoids negative substrate-to-source voltages of high-side transistors. Otherwise, the static depletion of the high-side channel (increasing the static on-resistance) during high-side conduction phases would be severe already at a low DC-operation voltage of below 50 V (measurements in [69]). Compared to GaN-on-SOI [19] or backsurface isolation using Si-based p–n junction isolation [20], which allows zero substrate-to-source voltage in all integrated transistors, this work’s pure GaN-on-Si approach cannot completely avoid dynamic electric fields between the source and substrate. Thus, electron trapping in the GaN buffer is still enhanced, which can result in dynamically increased on-resistance [10]. However, this effect is only significant at higher operation voltages. In [21,67], the dynamic on-resistance effect from the electric fields in the bridge circuits was observed only beyond 200 V, such that the low-voltage applications addressed in this work are not expected to significantly suffer from the dynamic on-resistance effect. Trapping mechanisms are discussed in [8].

Figure 10d shows the measured terminal capacitances (LCR-meter with bias-tees, 1 MHz, measurements between two terminals and AC-grounding other terminals) of one integrated half-bridge at $V_{\mathrm{DC}}=V_{B}=24\;V$, where each gate was short-circuited with the respective source (off-state). The resulting four terminals (1: switch-node and high-side gate SW, 2: substrate B, 3: negative DC-link and low-side gate DC-, 4: positive DC-link DC+) result in six measured terminal capacitances. Even though the semi-floating substrate potential around the positive DC-link voltage analyzed in paper is nonconstant over time (due to capacitive coupling), the capacitance measurement is only presented for a single and fixed substrate potential of $V_{B}=24$ V. The effect of the substrate bias dependence on the capacitances is negligible for the low operation voltages in this paper. In addition to the low operation voltage, the fact that most of the layout structure is metallized leads to a large portion of fixed capacitance, which also contributes to the low bias dependence of the capacitances. For the validity of later calculations based on the measured capacitances, it should be noted that the three integrated phase legs (half-bridges) are not electrically interconnected already on-chip. Each half-bridge has separate DC-link terminals and bond pads, such that the measured terminal capacitances connected to the DC-link terminals are the correct values of one half-bridge. The typically decreasing transistor output capacitance with increased drain–source voltage is visible in the half-bridge measurement as a decreasing $C_{\mathrm{SW}-\mathrm{DC}-}$ for the low-side (where $V_{\mathrm{DS},\mathrm{LS}}=V_{\mathrm{SW}}$) and an increasing $C_{\mathrm{SW}-\mathrm{DC}+}$ for the high-side (where $V_{\mathrm{DS},\mathrm{HS}}=-V_{\mathrm{SW}}+V_{\mathrm{DC}}$).

For the monolithic integrated three-phase circuit on semi-floating substrate, the expected transient substrate voltage follows from charge-conservation on the substrate $Q_{B}\left(t\right)=\mathrm{const}.$ (assuming $R_{\mathrm{SF}}\to \infty$) as presented in [62] as

(1) $$Q_{B}=3V_{B}\left(t\right)\left[C_{0}+C_{\mathrm{SW}}\right]-C_{\mathrm{SW}}\left[V_{\mathrm{PH}1}\left(t\right)+V_{\mathrm{PH}2}\left(t\right)+V_{\mathrm{PH}3}\left(t\right)\right],$$

where $C_{0}=C_{B-\mathrm{DC}-}+C_{B-\mathrm{DC}+}$ and $C_{\mathrm{SW}}=C_{B-\mathrm{SW}}$ form a capacitive divider circuit from the switch-nodes through the substrate to the DC-link, assuming an externally stabilized DC-link voltage by sufficiently large DC-link capacitors [70]. For simplicity, the following calculations are based on the averages (over $V_{\mathrm{SW}}$ from 0 to 24 V) of the measured terminal capacitance values. By sufficiently high resistance of the biasing resistor $R_{\mathrm{SF}}$, the RC time constant of this resistor and the substrate capacitances is significantly longer than a switching cycle for typical switching frequencies above 10 kHz, such that the capacitive coupling is not significantly superimposed by charging current through the resistor. Only on a slower timescale is the resistor effective to bias the average of the substrate voltage. The highly resistive substrate biasing was inspired by the work of B. Weiss (first high-voltage operation of monolithic half-bridge [66]). For a constant (externally provided) DC-link voltage, and four types of switching states (either HIGH or LOW for phase voltages of $V_{\mathrm{DC}}$ or 0) of the three-phase bridge (1/HHH: all bridges HIGH, 2/LHH: one bridge LOW, two bridges HIGH, 3/LLH: two bridges LOW, one bridge HIGH, 4/LLL: all bridges LOW), four discrete levels of substrate potential result. The maximum substrate voltage swing $\Delta V_{B}=V_{\mathrm{DC}}{\left(1+C_{0}/C_{\mathrm{SW}}\right)}^{-1}$ [62] is the difference between the highest (during HHH state) and lowest (during LLL state, assuming symmetric utilization of the zero-states by SVPWM) discrete substrate voltage level. Based on the measured capacitance data, this is $\Delta V_{B}\approx 0.43\;V_{\mathrm{DC}}$, which is $\Delta V_{B}\approx 10.38\;V$ for 24 V operation.

If no capacitance measurement data are available, $\Delta V_{B}$ can be estimated by geometry assumptions [62]: Assuming that the low-voltage design is limited by the design rules of the ohmic contacts, the length of each DC-link ohmic contact is equal to the length of the switch-node ohmic contact, and the channel length is negligible compared to the ohmic contact length, then the geometry assumption of $C_{0}/C_{\mathrm{SW}}=1/2$ results in a calculated $\Delta V_{B}=0.5\;V_{\mathrm{DC}}$ and $\Delta V_{B}\approx 12\;V$ for 24 V operation. It is noted that the maximum $\Delta V_{B}$ of the three-phase bridge is similar to the case of a monolithic half-bridge with semi-floating substrate [66], since hypothetical switching of all three-phases only between LLL and HHH states is similar to a half-bridge topology. The difference here, however, is that the additional switch states introduce additional discrete substrate levels. The four discrete substrate levels during three-phase operation are equally spaced by $\frac{1}{3}\Delta V_{B}$, resulting from Equation (1) by evaluation for the different switch states.

Biasing the substrate with a highly resistive substrate biasing resistor allows us to shift the average substrate potential to an arbitrary voltage. The biasing approach in this work is to the positive DC-link voltage to reduce negative back-gating with related possible static on-resistance increase of the high-side transistors during conduction phases. In the following, it is thus assumed that the average substrate is biased around $V_{\mathrm{DC}}$. For the LLL state, the substrate level is the lowest of the four levels ($V_{B}=V_{\mathrm{DC}}-\frac{1}{2}\Delta V_{B}$), but during this state no high-side transistor is conducting. The LLH state, where only one high-side transistor and two low-side transistors are conducting, is the state where the substrate voltage $V_{B}=V_{\mathrm{DC}}-\frac{1}{2}\Delta V_{B}+\frac{1}{3}\Delta V_{B}=V_{\mathrm{DC}}-\frac{1}{6}\Delta V_{B}$ is lowest during all times where at least one high-side transistor is conducting. This worst-case high-side substrate-to-source voltage during conduction phases is now calculated from the measured capacitances as −1.7 V (ratio of −7.2% from the DC-link voltage) or −2.0 V (ratio of −8.4%) with the assumed geometry ratio but without capacitance data.

Compared to two published semi-floating substrate biasing networks for GaN-on-Si half-bridges which had up to $-\frac{1}{2}V_{\mathrm{DC}}$ (Figure 10b left [66]), and with an improved circuit zero (Figure 10b right [69]) negative high-side back-gating, this work now enables low back-gating of $-\frac{1}{12}V_{\mathrm{DC}}$ for three-phase ICs (Figure 10a).

### 3.4. Measurement of Substrate Biasing during Motor Operation

**Figure 11:**
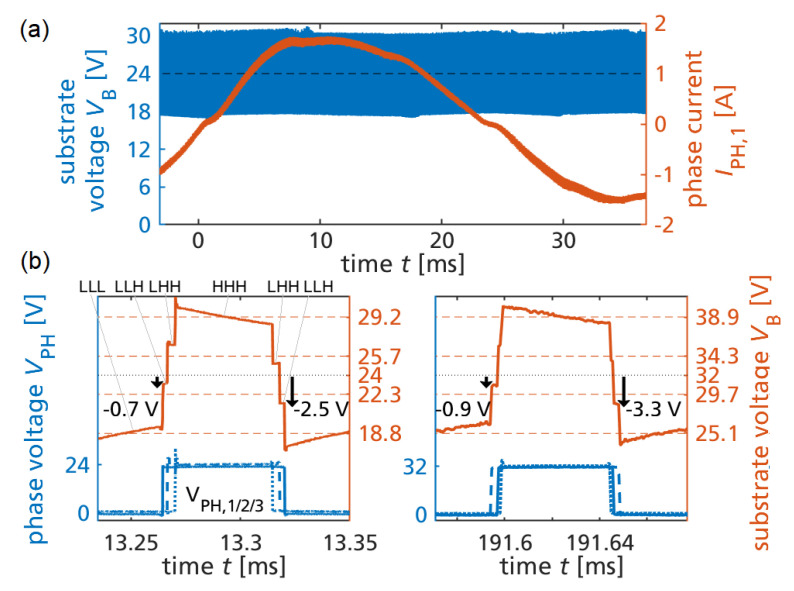
(**a**) Substrate voltage and phase current at 24 V motor drive. (**b**) Phase and substrate voltage during one switching cycle at 24 V and 32 V. Dashed: calculated substrate levels. Arrows: back-gating voltage of high-side on-state.

Figure 11a shows the measured current of one of the three phases and the substrate voltage over the cycle time of the sinusoidal current, which verifies that, on average, the substrate is DC-biased to 24 V. Figure 11b (left) shows a zoom during one switching cycle, where the switch states are labeled, and discrete substrate levels are visible in the common substrate voltage as a result of the capacitive coupling from the three switch node voltages (also shown). The highest and lowest measured voltage levels (solid) match the calculated levels (dashed lines). Since $R_{\mathrm{SF}}$ is not infinite, and the switching frequency is low (10 kHz), a slow exponential decay towards the 24 V bias is observed. Due to this superimposed signal, the two middle substrate voltages are slightly offset from the calculated values. The measured worst-case substrate-to-source voltages of high-side transistors during conduction in the LLH states are visible as two values (−2.5 V and −0.7 V), which, on average (−1.6 V), is close to the calculated value of −1.7 V. Figure 11b (right) shows additional measurements for an increased DC-link voltage of 32 V and the calculated substrate voltage levels. The measured back-gating (−0.9 V and −3.3 V) is now, on average, −2.1 V, still around −7% of the DC-link voltage, which verifies the proportionality to the DC-link voltage according to the previous analysis. The measurements verify that the intentional semi-floating substrate biasing allows us to predict the expected substrate potential levels based on measured substrate capacitance data and circuit analysis, or with a simplified approach using assumed geometry ratios without capacitance measurement data.

## 4. Discussion and Conclusions

Table 1 compares this work’s GaN IC to previously published GaN ICs, which have either integrated power stages suitable for motor drive, or integrated current sensing (using senseFET or shunt methods). This work, for the first time, demonstrates continuous motor drive operation with a GaN IC which integrates both a three-phase motor stage and integrated current sensing.

A monolithic low-voltage three-phase inverter IC with integrated current sensing can replace the six typically required discrete power transistors (or three monolithic GaN half-bridges) and external current shunt resistors by just one semiconductor device. The current sensor integration is nonintrusive and thus does not add circuit parasitic in the critical switched current loops (for example, to external series-shunt resistors). Since the current mirror transistors are just scaled GaN HEMTs, otherwise equally designed as the main power transistors, the switching speed of the sense-FET is not limited by the measurement principle. Theoretically, the same switching speed as the main power transistor could be achieved if the external readout amplifier is properly designed (or also integrated in the GaN IC, which was carried out by this work but not yet used). The fast measured 51 ns risetime of the current sensor signal (without intentional optimization for high bandwidth) demonstrates that around 7 MHz measurement bandwidth can be realized, which is otherwise difficult to achieve. The external transimpedance amplifier circuit was connected through the PCB and DIP adapter, which is not an optimal layout. With improved amplifier design, even higher bandwidth is expected. The DC-biased semi-floating substrate biasing enables predictable substrate potentials with four discrete levels during SVPWM. The DC-biasing avoids negative back-gating of high-side transistors during high-side conduction on the common conductive Si substrate.

Low-voltage integrated power topologies in a pure GaN-on-Si technology enable area-efficient and, thus, low-cost application, such as for motor drives.

## Figures and Tables

**Table 1:** Comparison of GaN power ICs with power stages and current sensing.

	This Work	Power Stage (Multi-Switch) Only			One Transistor Only + Sensing	
**GaN IC**	3-phase bridge + 3 senseFETs	3-phase, no sensing	3×3-matrix, no sensing	1-phase, 3-level, no sensing	HEMT + senseFET	HEMT + shunt
**Operation voltage**	32 V	100 V [25]	150 V [26,71]	300 V [27]	48 V [35,64], 50 V [32], 200 V [33]	10 V [39], 70 V [40]
**Sensor bandwidth**	7 MHz (52 ns)	no sensor	no sensor	no sensor	43 MHz [32], 9.2 MHz (38 ns) [35]	3.8 Mhz (90 ns) [40]
**Motor drive**	yes (3-phase BLDC)	yes (3-phase BLDC)	no (pulsed switching)	no (1-phase inductive load)	no	no
**Efficiency**	n/a	93% [25]	n/a	n/a	n/a	n/a
**Integrated sense amplifier**	no	no	no	no	no	yes (1 MHz) [41]

## Data Availability

Data are contained within the article.
